# Phenotypic and genotypic characterization of *Aeromonas hydrophila* isolated from freshwater fishes at Middle Upper Egypt

**DOI:** 10.1038/s41598-025-89465-4

**Published:** 2025-02-18

**Authors:** Usama H. Abo-Shama, Amany A. Abd El Raheem, Reem M. Alsaadawy, Haitham H. Sayed

**Affiliations:** 1 Department of Microbiology, Faculty of Veterinary Medicine, Sohag University, Sohag, 82524 Egypt; 2 Department of Zoonoses, Faculty of Veterinary Medicine, Assiut University, Assiut, 71526 Egypt

**Keywords:** *Aeromonas hydrophila*, Freshwater fishes, Genes, PCR, Resistance, Virulence, Microbiology, Molecular biology

## Abstract

*Aeromonas hydrophila* is a common fish pathogen and a significant foodborne pathogen of increasing public health concern. This study was conducted in Middle Upper Egypt to determine the prevalence of *A. hydrophila* among the diseased *Oreochromis niloticus* (*n* = 100) and *Clarias gariepinus* (*n* = 100) at Assiut and Sohag Governorates. *A. hydrophila* isolates (*n* = 44) were assessed for antimicrobial susceptibility and biofilm production. Moreover, PCR was performed to analyze the incidence of some genes in 20 isolates of *A. hydrophila*. The results showed that 24% and 20% of the examined *O*. *niloticus* and *C*. *gariepinus* were infected with *A. hydrophila* respectively, with all (100%) showing a variety of clinical signs of septicemia. *A. hydrophila* isolates were all biofilm producers, with varied degrees of biofilm production. 79.5% of the isolates were multidrug-resistant and had a high multiple antimicrobial resistance index > 0.2. PCR analysis revealed that all isolates carried *act* and *blaTEM* genes but not carried *int2* gene. Additionally, *sul1*,* aer*,* tetA*,* int1*, and *qnrA* genes were present in 75%, 60%, 55%, 55% and 45% of them, respectively. This study highlights the high incidence of multidrug-resistant pathogenic *A. hydrophila* in the infected fishes, posing a serious risk to humans and fish.

**Supplementary Information:**

The online version contains supplementary material available at 10.1038/s41598-025-89465-4.

## Introduction

Egypt is the world’s ninth-largest fish producer, with an annual production of 1.5 billion tons, of which approximately 79.6% is contributed by aquaculture^1^. Aquaculture and fisheries represent a significant sector of the Egyptian national income^2^, as fish is considered an important source of high-quality animal protein and a cheap alternative to red meat^3^. In recent years, great interest was directed to the Egyptian fish industry to meet the increasing demands for animal protein^4^. In this regard, *Oreochromis niloticus* (*O. niloticus*) and *Clarias gariepinus* (*C. gariepinus*) are the main species reared in aquaculture in Egypt^5^ and are considered the most widely consumed fish species^6^.

Bacterial diseases are the most common cause of morbidity and mortality among wild fish^7^, and they are the most important threat to aquaculture that cause heavy mortalities under stress conditions^8^. Egypt has experienced massive mortalities eruptions all over fish farms leading to serious economic losses and several studies denoted that they might occur due to the infection with septicemic bacterial pathogens under the adverse aquatic environmental conditions^1^.

*Aeromonas hydrophila* is a facultatively anaerobic, gram-negative, and oxidase-positive bacterium^9^. It is present in aquatic environments and as part of the fish’s normal flora^10,11^. However, being opportunistic, it can cause disease whenever the opportunity arises, either under stress conditions, along with the other pathogens, or due to decreased fish resistance to infection^12^. *A. hydrophila* is a significant bacterial fish pathogen causing Motile *Aeromonas* Septicemia (MAS) in both farmed and wild fish^13,14^. MAS is one of the most potent diseases that affect fish globally and poses a serious threat to Egypt and other fish industry countries^15^.

*Aeromonas’s* pathogenicity is multifaceted, depending on a variety of virulence factors (VFs) such as adhesions, cytotoxins, hemolysins, lipases, proteases, and the capacity of bacteria to form biofilms^16^. Exotoxins are major VFs of *Aeromonas* spp.^17^, and because *A. hydrophila* produces hemolysins, enterotoxins, and cytotoxins, it poses a considerable risk to human health^18^. Food biofilm development causes food-borne illnesses, food deterioration and rejection, and economic losses^19^.

*Aeromonas* spp. isolated from aquatic environments are showing an increase in antimicrobial resistance (AMR)^20^. Additionally, the frequent occurrence of multidrug resistance (MDR) *Aeromonas* spp. among freshwater fishes is raising concerns because of their possible role as reservoirs^21^. Integrons play a significant role in the dissemination of antimicrobial resistance genes (AMR genes) among various bacterial species and may develop multi-resistance. Although three classes of integrons are implicated in AMR, class 3 integrons are infrequently found in clinical isolates^22^, despite their well-known presence in fish-farming contexts^23^.

Several different *Aeromonas* spp. are responsible for the zoonotic disease aeromoniasis, which is contracted through direct contact with swimming water or by consuming infected fish, seafood, and drinking water^24^, and *A. hydrophila* is the most significant zoonotic pathogen to be concerned about^25^. *A. hydrophila* is a foodborne pathogen that has been relevant to public health concerns in recent decades^18^. Additionally, *A. hydrophila* can cause several illnesses in humans, such as endocarditis, osteomyelitis, septic arthritis, fulminating septicemia, meningitis, gastroenteritis, and a high mortality rate in immunocompromised persons^26^.

Numerous studies have investigated the occurrence, virulence, and AMR profile of *A. hydrophila* isolated from freshwater fish across various Governorates in Egypt^14,17,26–34^. However, to the best of our knowledge, limited research^24,35^ has focused on the prevalence, AMR profile, and virulence of *A. hydrophila* isolated from *O. niloticus* in Middle Upper Egypt. Therefore, using a combination of clinical and bacteriological examinations, the current study aimed to assess prevalence of *A. hydrophila* infection among the diseased *O. niloticus* and *C. gariepinus* in Assiut and Sohag Governorates, along with determining the isolates’ antimicrobial susceptibility (AMS), biofilm-forming capacity, and distribution of certain virulence factor genes (VF genes) (*act* and *aer*), AMR genes (*blaTEM*, *qnrA*, *sul1* and *tetA*) and class 1 and 2 integrons among the isolates by PCR. These data are essential to guide the appropriate choice of treatment options against *A. hydrophila* infection, and to put the right preventive and control measures to work for infections.

## Results

### Clinical and post-mortem findings

The infected fish displayed a variety of clinical signs of septicemia, such as hemorrhages on different body regions, loss of scales, skin ulceration, fin rot, and abdominal distension. Internally, there was hemorrhagic enteritis, liver congestion, enlargement, and paleness, in addition to reddish ascetic exudate.

### Phenotypic and genotypic characterization of *A*. *Hydrophila* from the examined fishes

*Aeromonas hydrophila* isolates generated small circular green colonies with a darker core on *Aeromonas* medium base (AMB), while generated round, smooth, yellowish, or creamy colonies on Tryptone Soya Agar (TSA). Microscopically, they were motile, gram-negative, short bacilli.

*Aeromonas hydrophila* isolates were biochemically similar, except for citrate and lactose utilization tests, where they were positive for oxidase, catalase, arginine dihydrolase, lysine decarboxylase, b-glucoronidase (bGL), β-galactosidase, *N*-acetylglucosaminidase, phosphatase, b-glukuronidase (GLK), aesculin hydrolysis, indole production, nitrate reduction, and utilization of glucose, sucrose, mannitol and trehalose. However, they were negative for urease, ornithine decarboxylase, phenylalanine deaminase, gamma glutamyl transferase, H_2_S production, and utilization of inositol, sorbitol, rhaminose, raffinose, adonitol, dulcitol, celliobiose and malonate. Genotypically, the isolates (*n* = 44) were confirmed to be *A. hydrophila* by detecting the *16 S rRNA* gene of the species (Supplementary Fig. 1).

Out of 200 examined samples, *A. hydrophila* was isolated from 44 samples (22%). *A. hydrophila* infection prevalence in the freshwater fish under investigation was compiled in Table 1. Fish was considered infected with *A. hydrophila*, if it was isolated from at least one organ. The isolation rate of *A. hydrophila* from the internal organs of the examined fishes is illustrated in Table 2.

**Table 1:** Prevalence of *A. hydrophila* infection among the examined freshwater fishes.

Governorate	Number of examined fish samples		Number of *O. niloticus* samples infected with *A. hydrophila*		Number of *C. gariepinus* samples infected with *A. hydrophila*	
	*O. niloticus*	*C. gariepinus*	No.	%	No.	%
Assuit	50	50	13	26	9	18
Sohag	50	50	11	22	11	22
Total	100	100	24	24	20	20

**Table 2:** Isolation rate of *A. Hydrophila* from the internal organs of the examined fishes.

Fish species	Liver		Kidney		Spleen	
	No.	%	No.	%	No.	%
*O. niloticus*	24	24	20	20	12	12
*C. gariepinus*	18	18	15	15	11	11
Total	42	21	35	17.5	23	11.5

In terms of biofilm formation, it was found that 27.3% (*n* = 12), 52.3% (*n* = 23), and 20.4% (*n* = 9) of the isolates were strong, moderate, and weak biofilm producers (BPs), respectively.

### Antimicrobial susceptibility profile of *A. hydrophila* isolates

*Aeromonas hydrophila* isolates were resistant to Amoxicillin (AX), Cephalothin (KF), Oxytetracycline (T), Erythromycin (E), Nitrofurantoin (F), Amoxicillin/clavulanic acid (AMC) and Nalidixic acid (NA), while were susceptible to Imipenem (IPM), Colistin (CT), Amikacin (AK), Chloramphenicol (C), Ciprofloxacin (CIP), Gentamicin (CN), Norfloxacin (NOR) and Trimethoprim/ Sulphamethoxazole (SXT) (Table 3). Furthermore, 79.5% of the isolates were multidrug resistant (MDR) with variable multiple antimicrobial resistance (MAR) index ranging from 0.133 to 0.933 and they included (19) different AMR patterns (Table 4). There was a significant association between phenotypic AMR and biofilm production in four of the studied antimicrobial agents (Supplementary Table 1).

**Table 3:** Antimicrobial susceptibility results of *A. hydrophila* isolates.

Antimicrobial class	Antimicrobial agent	Results of *A. hydrophila* isolates (*n* = 44)					
		Sensitive		Intermediate		Resistant	
		No.	%	No.	%	No.	%
β-lactams	AX	0	0	0	0	44	100
	AMC	7	15.9	3	6.8	34	77.3
	KF	0	0	0	0	44	100
	IPM	40	90.9	3	6.8	1	2.3
Aminoglycosides	CN	28	63.7	6	13.6	10	22.7
	AK	29	65.9	7	15.9	8	18.2
Macrolides	E	4	9.1	5	11.4	35	79.5
Quinolones	CIP	31	70.4	5	11.4	8	18.2
	NOR	29	65.9	5	11.4	10	22.7
	NA	14	31.8	7	15.9	23	52.3
Sulfonamides	SXT	19	43.1	5	11.4	20	45.5
Nitrofurans	F	5	11.4	4	9.1	35	79.5
Phenicols	C	26	59.1	10	22.7	8	18.2
Polymyxins	CT	38	86.4	2	4.5	4	9.1
Tetracyclines	T	5	11.4	2	4.5	37	84.1

**Table 4:** Antimicrobial resistance profile of *A. hydrophila* isolates (*n* = 44).

Antimicrobial resistance pattern (*n* = 19)	Isolates of this resistance pattern		Number of resistant antimicrobials	Number of resistant antimicrobials classes	MAR index
	No.	%			
AX, KF, T, E, F, AMC, NA, SXT, CN, NOR, CIP, AK, C, CT	1	2.3	14	9	0.933
AX, KF, T, E, F, AMC, NA, SXT, CN, AK, C, CT, IPM	1	2.3	13	9	0.867
AX, KF, T, E, F, AMC, NA, SXT, CN, NOR, CIP, AK, C	2	4.5	13	8	0.867
AX, KF, T, E, F, AMC, NA, SXT, CN, NOR, CIP, AK	1	2.3	12	7	0.800
AX, KF, T, E, F, AMC, NA, SXT, CN, NOR, C	1	2.3	11	8	0.733
AX, KF, T, E, F, AMC, NA, SXT, NOR, CIP, C	1	2.3	11	7	0.733
AX, KF, T, E, F, AMC, NA, SXT, CN, AK	3	6.8	10	7	0.667
AX, KF, T, E, F, AMC, NA, SXT, CN, NOR	1	2.3	10	7	0.667
AX, KF, T, E, F, AMC, NA, SXT, NOR, CIP	1	2.3	10	6	0.667
AX, KF, T, E, F, AMC, NA, NOR, CIP	1	2.3	9	5	0.600
AX, KF, T, E, F, AMC, NA, SXT	6	13.6	8	6	0.533
AX, KF, T, E, F, AMC, NA, CT	2	4.5	8	6	0.533
AX, KF, T, E, F, AMC, NA, C	2	4.5	8	6	0.533
AX, KF, T, E, F, AMC, NOR, CIP	1	2.3	8	5	0.533
AX, KF, T, E, F, AMC, SXT	1	2.3	7	5	0.467
AX, KF, T, E, F, SXT	1	2.3	6	5	0.400
AX, KF, T, E, F, AMC	9	20.5	6	4	0.400
AX, KF, T	2	4.5	3	2	0.200
AX, KF	7	15.8	2	1	0.133

### Distribution of antimicrobial resistance, virulence, and integrons genes among *A. hydrophila* isolates

Table 5 and Supplementary Fig. 2 through 9 show PCR results for the genes that were examined in *A. hydrophila* isolates in this investigation. Furthermore, Table 5 demonstrates the relationship between AMR phenotypes and genotypes in the studied isolates. Statistical analysis revealed no significant associations between phenotypic and genotypic AMR (Supplementary Table 2). Additionally, there was a significant association between the genotypic AMR and biofilm production in *sul1* and *tetA* AMR genes (Supplementary Table 3). The class 1 and 2 intergrons and genotypic AMR association was presented in Supplementary Tables 4 and 5.

**Table 5:** Distribution of virulence, antimicrobial resistance, and integrons genes among the investigated *A. hydrophila* isolates (*n* = 20).

Isolate No.	Antimicrobial resistance pattern	Biofilm formation	Results of investigated genes							
			act	aer	blaTEM	qnrA	sul1	tetA	int1	int2
A1	AX, KF, T, E, F, AMC, NA, SXT, CN, NOR, CIP, AK, C, CT	+	+	-	+	+	+	+	+	−
A2	AX, KF, T, E, F, AMC, NA, SXT, CN, AK, C, CT, IPM	+	+	+	+	−	+	+	+	−
A3	AX, KF, T, E, F, AMC, NA, SXT, CN, NOR, CIP, AK, C	+	+	+	+	+	+	−	+	−
A4	AX, KF, T, E, F, AMC, NA, SXT, CN, NOR, CIP, AK	+	+	−	+	+	−	+	+	−
A5	AX, KF, T, E, F, AMC, NA, SXT, CN, NOR, C	+	+	−	+	−	+	−	−	−
A6	AX, KF, T, E, F, AMC, NA, SXT, NOR, CIP, C	+	+	+	+	+	+	+	+	−
A7	AX, KF, T, E, F, AMC, NA, SXT, CN, AK	+	+	+	+	−	−	+	−	−
A8	AX, KF, T, E, F, AMC, NA, SXT, CN, NOR	+	+	+	+	+	+	+	+	−
A9	AX, KF, T, E, F, AMC, NA, SXT, NOR, CIP	+	+	+	+	−	+	−	+	−
A10	AX, KF, T, E, F, AMC, NA, NOR, CIP	+	+	−	+	−	+	−	−	−
A11 & A12	AX, KF, T, E, F, AMC, NA, SXT	+	+	+	+	+	+	+	+	−
A13	AX, KF, T, E, F, AMC, NA, CT	+	+	+	+	−	−	−	+	−
A14	AX, KF, T, E, F, AMC, NA, C	+	+	−	+	−	+	−	−	−
A15	AX, KF, T, E, F, AMC, NOR, CIP	+	+	−	+	+	−	−	+	−
A16	AX, KF, T, E, F, AMC, SXT	+	+	+	+	−	+	+	−	−
A17	AX, KF, T, E, F, SXT	+	+	+	+	+	+	+	−	−
A18	AX, KF, T, E, F, AMC	+	+	−	+	−	+	+	−	−
A19	AX, KF, T, E, F, AMC	+	+	−	+	−	+	−	−	−
A20	AX, KF, T, E, F, AMC	+	+	+	+	−	−	−	−	−
Total		100	100	60	100	45	75	55	55	0

## Discussion

The current investigation provided important data regarding the prevalence, virulence, and AMR of *A. hydrophila* infecting *O. niloticus* and *C. gariepinus* in Middle Upper Egypt, together with the distribution of class 1 and 2 integrons among *A. hydrophila* isolates. The studied fish samples exhibited clinical signs and post-mortem lesions that were similar to those previously documented^17,29^. These findings could be attributed to bacterial invasion and colonization, as well as the action of toxic extracellular products of *A. hydrophila*, such as aerolysin and cytotoxic enterotoxin^36^.

Establishing successful control of fish bacterial infections and avoiding the associated substantial economic losses and health concerns for humans depends mainly on accurate diagnosis^37^. Phenotyping, serology, and genotyping are employed in combination to identify bacterial pathogens^38^. Moreover, the isolation and identification of *Aeromonas* spp. are based on the colonial features on the selective media and outcomes of the biochemical tests^29^. All of the isolates in this study showed typical morphological and biochemical characteristics of *A. hydrophila* almost identical to those previously reported^15,39–42^. However, *A. hydrophila* isolates showed different results in the consumption of lactose and citrate, which can be explained by the variations in the origin and fish species^10^, and by the presence or lack of genes that regulate metabolic traits^7^. While phenotyping, which relies on biochemical testing, is widely employed to identify bacteria, genotyping is a more reliable method due to the inconsistent results it can yield sometimes^40^. When combined with biochemical assays, the *16 S rRNA* gene is a significant diagnostic tool for microbes identification^43^. All the isolates in our investigation were genotypically verified as *A. hydrophila* by detection of *16 S rRNA* gene by PCR. Although similar findings were previously reported^18,28,44^, our results differed from those of one study^23^, which did not detect the *16 S rRNA* gene in 57% of the phenotypically identified *A. hydrophila* isolates from rainbow trout farms in Australia. This can be explained by the different primers used to target the *16 S rRNA* gene, where microheterogeneity in *16 S rRNA* gene sequence within the bacterial species is common^23^, or by the size of the primers used since the size of the amplified *16 S rRNA* gene fragment can affect the sensitivity of PCR for *16 S rRNA* gene^45^.

The results presented in this study indicated that *A. hydrophila* was present in 26% and 22% of *O. niloticus* samples examined in Assuit and Sohag Governorates, Egypt, respectively, with a total prevalence of 24%. Similarly, 18% and 22% of *C. gariepinus* samples examined in these Governorates were infected with *A*. *hydrophila*, with a total prevalence of 20%. These findings suggest that *A. hydrophila* is one of the common fish pathogens in Middle Upper Egypt and poses a health risk to consumers. Additionally, the prevalence of *A. hydrophila* infection was higher among *O. niloticus* than *C. gariepinus*. These findings were almost identical to those previously reported by many studies in Egypt^45–47^. However, they were contrary to the findings reported by other studies^17,30,48^, and this disagreement might be explained by the variations in water quality, sampling season, management techniques, and/or geographic location^14^.

Bacteria create structures called biofilms that increase their pathogenic potential, virulence, and resistance to antimicrobials (AMs), as well as their tendency to cause persistent infections^14^. All *A. hydrophila* isolates in the current study were found to be BPs but to varying degrees regarding biofilm formation. Our findings corroborated those of Bakhtiari et al.^49^; however, Maarouf et al.^50^ reported that only 76.7% of *A. hydrophila* isolates were BPs.

Virulence of *Aeromonas* spp. is primarily dependent on their exotoxin release^51^. Detection of *aer*,* act*,* lip*,* ser*, and *eprCAI* genes has been widely used for assessing *Aeromonas* species’ potential pathogenicity^39^. Cytotoxic enterotoxin is the most cytotoxic VFs within *A. hydrophila*, and it has multiple effects including cytotoxic, cytotonic, and hemolytic activities^16^. It is also one of the main VFs associated with human gastroenteritis^14^. *Act* gene was present in all *A. hydrophila* isolates in this study, which is consistent with previous findings^15,39,52^. In contrast, other studies reported that the *act* gene was present in only 60%, 37.5%, and 18.18% of *A. hydrophila* isolates, respectively^17,28,46^.

One of the primary VFs causing gastroenteritis in humans, aerolysin encoded by *Aer* gene, plays a key role in the pathogenesis of *A. hydrophila* infection in fish^28^. It has hemolytic, cytotoxic, and enterotoxic properties^53^. The present investigation revealed that 60% of *A. hydrophila* isolates carried *aer* gene, which is consistent with findings of previous studies^28,54^. In contrast, the findings of Eid et al.^55^ and Salem et al.^36^ indicated that 100% and 83.3% of *A. hydrophila* isolates carried *aer* gene respectively.

Following Deng et al.^56^, *Aeromonas* spp. have become resistant to a variety of AMs, and the prevalence of MDR *Aeromonas* spp. is rising globally^57^. As detected in the current study, *A. hydrophila* isolates were resistant to AX and KF followed by T, E, F, AMC, and NA, while they were sensitive to IPM, CT, AK, C, CIP, CN, NOR, and SXT. Previous research reported nearly identical results except for the isolates’ susceptibilities to T and NA and SXT respectively^32,46^. Contrary to our findings, Saleh et al. reported that *A. hydrophila* isolates were resistant to AK and C, but sensitive to AMC and T^30^. This difference in susceptibility to management practices, geographic location, frequency, length, and amount of AMs use, and/or AMS method can be explained.

In line with findings of the previous literature, 79.5% of *A. hydrophila* isolates exhibited MDR and had a high MAR Index ˃ 0.2 ^32,36,47^. However, higher (100%) and lower (47.6%) prevalence of MDR *A. hydrophila* were previously reported^26,30^. The high prevalence of MDR *A. hydrophila* and high MAR index found in this study could be attributed to the indiscriminate use of AMs in the study area. Although some AMs were approved by the authority of veterinarian pharmaceuticals for aquaculture in highly producing countries^58^, in Egypt, AMs are frequently used in aquaculture without veterinary prescriptions, often for disease prevention and as growth promoters, contributing to the development and spread of resistance^52^. Additionally, contamination of the aquatic environment by AMs and their resistance genes from other sources, such as treated wastewater effluents, untreated poultry manure that is used as pond fertilizer before stocking, and agricultural drainage contribute severely to this issue^15,59^. All of this poses a threat as it will make treatment of *A. hydrophila* infections in aquaculture more difficult or even impossible, and it will also have detrimental effects on the environment and public health. Consequently, the non-therapeutic use of AMs must be prohibited, and AMS should be conducted before therapy to ensure effective treatment. Moreover, strict biosecurity measures must be applied to mitigate the spread of resistance.

In aquaculture and agriculture, six common classes of AMs, which are included in WHO list of AMs that are critically important for humans are frequently used. They include aminoglycosides, macrolides, penicillins, quinolones, sulphonamides, and tetracyclines^60^. On the other hand, several recently discovered genetic elements and resistance determinants for quinolones, tetracyclines, and β-lactamases were shown to be shared between aquatic bacteria, fish, and human pathogens and appear that they originated in aquatic bacteria^61^. In the current study, 20 representatively selected MDR *A. hydrophila* isolates were analyzed by PCR for the presence of *blaTEM*,* qnrA*,* sul1*, and *tetA* genes. The results revealed that these genes were detected in 100%, 45%, 75%, and 55% of these isolates, respectively. Our findings were in agreement with those of Abd El-Tawab et al., who reported that 100% and 80% of *A. hydrophila* isolates harbored *blaTEM* and *sul1* genes, respectively^48^, and also similar to those of Hossain et al., who reported that 50% of *A. hydrophila* isolates possessed *tetA* gene^62^. However, our findings were opposed to those of Ali et al., who found that none of the *A. hydrophila* isolates harbored *blaTEM* nor *sul1* genes^52^, and those of Ebied et al., who reported that all the isolates harbored *qnrA*,* sul1* and *tetA* genes^63^. The difference in these findings could be attributed to the geographical dispersion of the strain and the variation in contamination levels^64^.

On the other hand, analysis the relation between AMR phenotypes and genotypes in *A. hydrophila* isolates revealed that *blaTEM* gene was present in all β-lactams resistant isolates while *tetA*,* qnrA*, *qnrA*,* qnrA* and *sul1* genes were not detected in 45%, 35%, 15%, 10% and 10% of T, NA, NOR, CIP and SXT-resistant isolates, respectively, and this resistance could be attributed to another *tet*,* qnr* and *sul* gene (s) that aren’t examined in this study. Additionally, it revealed that 20%, 20%, 15%, 10%, and 5% of the investigated isolates were sensitive to CIP, SXT, NOR, NA, and AMC respectively although they harbored *qnrA*, *sul1*, *qnrA*,* qnrA*, and *blaTEM* genes. This discrepancy could be explained by the isolates’ lack of expression of these genes in vitro.

According to Rowe-Magnus et al., the fast evolution of MDR across many gram-negative bacteria is caused by the capture and distribution of AMR genes via integrons^65^. The occurrence of integrons in fish-farming environments is well-documented^23^, as there is an association with various resistance gene cassettes^66^. However, little is known about the occurrence of class 1 and 2 integrons in *A. hydrophila* in Egypt. Results of this study showed that class 1 integrons were present in 55% of *A. hydrophila* isolates, which is almost in concurrence with findings of the previous study^67^, which reported that 50% of *A. hydrophila* isolates harbored class 1 integrons. On the other hand, many studies reported that 18.2%, 16.7%, and 12.16% of *A. hydrophila* isolates harbored class 1 integrons, respectively^56,68,69^ and this difference can be attributed to the divergent sources of the isolates as well as the unique background of AMs usage^56^. Additionally, in line with the previous literature^67,68^, class 2 integrons weren’t found in all *A. hydrophila* isolates.

According to our findings, class 1 integrons are important for conferring MDR in *A. hydrophila.* They may also play a role in spreading MDR genes to various fish pathogens and possibly humans, which could complicate antimicrobial therapy in the future. This is where integrons are known to carry many AMR genes^66^ and numerous combinations of them have been reported within them. Additionally, these integrons could be horizontally transferred among the different species of bacteria, which is made easier and faster by their location on plasmids and transposons^22^.

## Conclusion

The current study demonstrated that there is a high incidence of MDR pathogenic *A. hydrophila* among *O. niloticus* and *C. gariepinus* in Assuit and Sohag Governorates, Egypt. Moreover, in addition to class 1 integrons, several *A. hydrophila* isolates had multiple AMR genes and VF genes, posing a serious risk to fish, humans, animals, and the environment. The indiscriminate use of AMs must be immediately restricted, and effective biosecurity and preventive management techniques should be implemented. Future studies should focus on the prevalence of MDR pathogenic *A. hydrophila* in various freshwater fish species, as well as on conducting epidemiological investigations of *A. hydrophila* infections among fishermen.

## Methods

### Ethical approval

The protocol of this study was approved by the Research Ethics Committee of the Faculty of Veterinary Medicine, Sohag University, Egypt (No. Soh.Un.Vet./00028 R1). All procedures were carried out according to the relevant guidelines and regulations. The study was conducted following the ARRIVE (Animals in Research: Reporting In Vivo Experiments) criteria^70^.

### Samples collection

A total of 100 samples were randomly collected from each of the clinically diseased *O. niloticus* and *C. gariepinus* (exhibiting symptoms consistent with septicemia) across various locations in Assiut and Sohag governorates, Egypt. The samples included live, moribund, and recently deceased fish were immediately sent to the laboratory within 1–2 h. Live fish were transported in a compact tank with an adequate volume of water with constant aeration, while deceased fish were placed in an insulated icebox maintained at 4–8 °C.

### Clinical and post-mortem examination

The euthanasia of the fish was done using an overdose of tricaine methane sulfonate (MS222-Sigma-Aldrich, Saint Louis, Missouri, USA)^71^. Briefly, a stock solution of the tricaine with a concentration of 10 g/L was prepared by adding sodium bicarbonate till saturation (final pH value 7.0–7.5). The stock solution was stored in the refrigerator in a dark bottle till use. For euthanasia, the fish was left in a tricaine solution of a concentration ≥ 250 mg/L for about 10 min following cessation of opercular movement. Following euthanasia, clinical and post-mortem examinations were performed on fish samples as previously described^58^, and all internal and external clinical abnormalities were documented.

### Isolation and phenotypic identification of *A. hydrophila*

Bacterial swabs were taken from the liver, kidney, and spleen of every fish under examination. After being enriched for 24 h at 30 °C in an aerobic environment in Tryptone Soya Broth (TSB) (Oxoid, Basingstoke, Hampshire, UK), they were streaked onto Tryptone Soya Agar (TSA) (Oxoid, Basingstoke, Hampshire, UK) and incubated for another 24 h at 30 °C in an aerobic environment^52^. Subsequently, the colonies that appeared yellow or creamy while growing on TSA were chosen, refined, examined under a microscope following gram staining, and tested for oxidase and catalase.

AMB (Oxoid, Basingstoke, Hampshire, UK) supplemented with ampicillin supplement (Oxoid, Basingstoke, Hampshire, UK) was then streaked with pure single colonies of gram-negative, oxidase-positive and catalase-positive bacilli isolates and incubated aerobically for 24 h at 30 °C^10^. Ultimately, probable *Aeromonas* colonies were chosen and biochemically identified using SRO GN 24 kit (Diagnostics s.r.o., Galanta, Slovakia) following the manufacturer’s instructions and Buller’s criteria^43^. All *A. hydrophila* isolates were stored at -80 °C in TSB containing 15% glycerol (Fluka, Seelze, Germany), and only one isolate was randomly selected from each positive sample and used for subsequent investigations.

### Biofilm formation assay

The microtiter plate method was used as previously described^14^. *A. hydrophila* isolates were cultured in TSB (Oxoid, UK) at 37 °C for 24 h. Afterward, they were diluted 1:100 in new TSB including 0.5% glucose (Piochem, 6th of October City, Giza, Egypt). Each *A. hydrophila* isolate was tested in triplicate by putting 100 µl of its diluted broth in three wells of a microtiter plate (Costar, USA) and three wells were created as negative controls, containing 100 µl of TSB and 0.5% glucose only.

The inoculated microtiter plate was then incubated for 24 h at 37 °C. Following that, the wells’ contents were removed, and phosphate-buffered saline (PBS) (Piochem, 6th of October City, Giza, Egypt) was used three times to thoroughly clean them. Following the microtiter plate’s drying, 200 µl of 1% (w/v) crystal violet stain (Fluka, Seelze, Germany) was applied to each well and allowed to sit at room temperature for one hour. Following removal of the stain and three thorough cleanings with PBS, each well received 200 µl of ethyl alcohol 95% (v/v) (EL Naser, Giza, Egypt). The microtiter plate was then incubated for 15 min at 25 °C.

The adhering biofilms’ optical densities (ODs) were determined at 620 nm using an ELISA plate reader (BioTek ELX800, Santa Clara, CA, USA). Based on OD, *A. hydrophila* isolates were interpreted as non-biofilm producers when OD test < OD control, weak BPs when OD control < OD test < 2 OD control, moderate BPs when 2 OD control < OD test < 4 OD control, and as strong BPs when OD test > 4 OD control.

### Antimicrobial susceptibility testing for *A. hydrophila* isolates

Kirby-Bauer method was used to assess the susceptibility of *A. hydrophila* isolates to AMs listed in Table 6 (Bioanalyse, Yenimahalle, Ankara, Turkey) on Mueller-Hinton agar plates (Oxoid, Basingstoke, Hampshire, UK). The test was performed and evaluated in compliance with Clinical and Laboratory Standards Institute^72^. *A. hydrophila* isolate was classified as MDR if it was resistant to at least three AMs of different classes^73^. MAR index was calculated for each isolate by dividing the number of AMs to which the isolate was resistant by the number of tested AMs. As previously reported, the MAR index value is often > 0.2 when bacteria are frequently exposed to several AMs, whereas it is < 0.2 when bacteria are seldom or never exposed to AMs^74,75^.

**Table 6:** Antimicrobials used in antimicrobial susceptibility of *A. Hydrophila* isolates.

Antimicrobial agent	Concentration of antimicrobial
Amoxicillin	10 µg
Amoxicillin/clavulanic acid	20/10 µg
Cephalothin	30 µg
Imipenem	10 µg
Gentamicin	10 µg
Amikacin	30 µg
Erythromycin	15 µg
Ciprofloxacin	5 µg
Norfloxacin	10 µg
Nalidixic acid	30 µg
Trimethoprim/sulphamethoxazole	1.25/23.75 µg
Nitrofurantoin	300 µg
Chloramphenicol	30 µg
Colistin	10 µg
Oxytetracycline	30 µg

### Genotypic characterization of *A. hydrophila* isolates

*16 S rRNA* gene of *A. hydrophila* was targeted by a PCR assay in this study to confirm the isolates genotypically. After that, 20 representively chosen MDR isolates were examined for the presence of specific genes, including integrons genes (*int1* and *int2*), AMR genes (*blaTEM*,* qnrA*,* sul1*, and *tetA*), and VF genes (*act* and *aer*). The utilized oligonucleotide primers (Metabion, Planegg, Germany) are shown in Table 7.

**Table 7:** Target genes, oligonucleotide primers, and PCR conditions used in the study.

Target gene	Primers sequences (5′–3′)	Product size (bp)	Primary denaturation	Number of cycles (No.) PCR conditions				Final extension	References
				No.	Denaturation	Annealing	Extension		
*16 S rRNA*	GAAAGGTTGATGCCTAATACGTA	625	94 °C 5 min	35	94 °C 30 s	50 °C 40 s	72 °C 45 s	72 °C 10 min	Abd El-Tawab et al.^28^
	CGTGCTGGCAACAAAGGACAG								
*act*	AGAAGGTGACCACCACCAAGAACA	232	94 °C 5 min	35	94 °C 30 s	55 °C 40 s	72 °C 30 s	72 °C 10 min	Eid et al.^15^
	AACTGACATCGGCCTTGAACTC								
*aer*	CACAGCCAATATGTCGGTGAAG	326	94 °C 5 min	35	94 °C 30 s	52 °C 40 s	72 °C 40 s	72 °C 10 min	Abd El-Tawab et al.^28^
	GTCACCTTCTCGCTCAGGC								
*blaTEM*	ATCAGCAATAAACCAGC	516	94 °C 5 min	35	94 °C 30 s	54 °C 40 s	72 °C 45 s	72 °C 10 min	Eid et al.^15^
	CCCCGAAGAACGTTTTC								
*qnrA*	ATTTCTCACGCCAGGATTTG	516	95 °C 4 min	30	95 °C 30 s	51 °C 30 s	72 °C 45 s	72 °C 10 min	Ebied et al.^63^
	GATCGGCAAAGGTTAGGTCA								
*sul1*	CGGCGTGGGCTACCTGAACG	433	94 °C 5 min	35	94 °C 30 s	60 °C 40 s	72 °C 45 s	72 °C 10 min	Eid et al.^15^
	GCCGATCGCGTGAAGTTCCG								
*tetA*	GGTTCACTCGAACGACGTCA	576	94 °C 5 min	35	94 °C 30 s	50 °C 40 s	72 °C 45 s	72 °C 10 min	
	CTGTCCGACAAGTTGCATGA								
*int1*	CAGTGGACATAAGCCTGTTC	160	94 °C 5 min	35	94 °C 30 s	56 °C 30 s	72 °C 1 min	72 °C 7 min	Hayati et al.^67^
	CCCGAGGCATAGACTGTA								
*int2*	GTAGCAAACGAGTGACGAAATG	788	94 °C 5 min	35	94 °C 30 s	56 °C 30 s	72 °C 1 min	72 °C 7 min	
	CACGGATATGCGACAAAAAGGT								

### DNA extraction

Following the manufacturer’s instructions, DNA was extracted from the purified isolates using the QIAamp DNA Mini Kit (QIAGEN Inc., Germantown, MD, USA). Nano Drop 1000 (Thermo Fisher Scientific, Wilmington, DE, USA) was used to quantify genomic DNA templates, which were then kept at −20 °C until used.

### DNA amplification

Targeted genes were amplified by PCR in a T3 thermocycler (Biometra, Jena, Germany) using EmeraldAmp GT PCR Master Mix (Takara, Kusatsu, Shiga, Japan) and PCR conditions listed in Table 7 for each gene. The manufacturer’s instructions were followed to create a PCR reaction mixture in 25 µl quantities.

### PCR product analysis

PCR products were screened by electrophoresis in 1.5% agarose gel (Biometra, Jena, Germany) using a 100 bp DNA ladder gene ruler (Thermo Fisher Scientific, Vilnius, Lithuania). The gel was then photographed using a gel documentation system (Alpha Innotech, San Leandro, CA, USA), and data was analyzed.

### Statistical analysis

In the current study, *A. hydrophila* prevalence, AMR of the *A. hydrophila* isolates, and distribution of the different genes among them were all represented as percentages. The association between biofilm production, phenotypic AMR, genotypic AMR, and integrons were assessed by the Chi-square test using SPSS version 27. P value < 0.05 were considered as statistically significant.

## Electronic supplementary material

Below is the link to the electronic supplementary material.

Supplementary Material 1

## Data Availability

The datasets used and/or analyzed during the current study are available from the corresponding author upon reasonable request.
