# Supplementary material for: Phenotypic and genotypic characterization of Aeromonas hydrophila isolated from freshwater fishes at Middle Upper Egypt
Source: Sci Rep. 2025 Feb 18;15:5920. doi: 10.1038/s41598-025-89465-4 (PMC11836066; doi:10.1038/s41598-025-89465-4)
Supplement: Supplementary file 1 — Supplementary Material 1 [file 41598_2025_89465_MOESM1_ESM.docx]

**Supplementary Table 1: Phenotypic antimicrobial resistance and biofilm production association (n=44)**

| **Antimicrobial Agent** | **Phenotypic Resistance** | **Biofilm production** | | | | **p value** |
| --- | --- | --- | --- | --- | --- | --- |
|  |  | **Strong biofilm producers (n)** | **Moderate biofilm producers (n)** | **Weak biofilm producers (n)** | **Non-biofilm producers (n)** |  |
| Amoxicillin | Sensitive  Intermediate  Resistant | 0  0  12 | 0  0  23 | 0  0  9 | 0  0  0 | - |
| Amoxicillin/clavulanic acid | Sensitive  Intermediate  Resistant | 0  1  11 | 3  2  18 | 4  0  5 | 0  0  0 | 0.082 |
| Cephalothin | Sensitive  Intermediate  Resistant | 0  0  12 | 0  0  23 | 0  0  9 | 0  0  0 | - |
| Imipenem | Sensitive  Intermediate  Resistant | 9  2  1 | 23  0  0 | 8  1  0 | 0  0  0 | 0.151 |
| Gentamicin | Sensitive  Intermediate  Resistant | 4  2  6 | 15  4  4 | 9  0  0 | 0  0  0 | **0.022** |
| Amikacin | Sensitive  Intermediate  Resistant | 4  3  5 | 18  2  3 | 7  2  0 | 0  0  0 | **0.043** |
| Erythromycin | Sensitive  Intermediate  Resistant | 0  0  12 | 2  3  18 | 2  2  5 | 0  0  0 | 0.168 |
| Ciprofloxacin | Sensitive  Intermediate  Resistant | 5  3  4 | 18  2  3 | 8  0  1 | 0  0  0 | 0.122 |
| Norfloxacin | Sensitive  Intermediate  Resistant | 4  3  5 | 17  2  4 | 8  0  1 | 0  0  0 | 0.068 |
| Nalidixic acid | Sensitive  Intermediate  Resistant | 3  0  9 | 7  4  12 | 4  3  2 | 0  0  0 | 0.134 |
| Trimethoprim/ Sulphamethoxazole | Sensitive  Intermediate  Resistant | 2  1  9 | 10  3  10 | 7  1  1 | 0  0  0 | 0.055 |
| Nitrofurantoin | Sensitive  Intermediate  Resistant | 0  0  12 | 2  3  18 | 3  1  5 | 0  0  0 | 0.086 |
| Chloramphenicol | Sensitive  Intermediate  Resistant | 8  1  3 | 9  9  5 | 9  0  0 | 0  0  0 | **0.016** |
| Colistin | Sensitive  Intermediate  Resistant | 9  2  1 | 21  0  2 | 8  0  1 | 0  0  0 | 0.228 |
| Oxytetracycline | Sensitive  Intermediate  Resistant | 0  0  12 | 4  0  19 | 1  2  6 | 0  0  0 | **0.032** |

**Supplementary Table 2: Phenotypic-genotypic antimicrobial resistance association (n=20)**

| **Antimicrobial Agent** | **Genotypic Resistance** | **Phenotypic Resistance** | | | **p value** |
| --- | --- | --- | --- | --- | --- |
|  |  | **Sensitive (n)** | **Intermediate (n)** | **Resistant (n)** |  |
| Amoxicillin | *BlaTEM* (+) | 0 | 0 | 20 | - |
|  | *BlaTEM* (-) | 0 | 0 | 0 |  |
| Amoxicillin/clavulanic acid | *BlaTEM* (+) | 0 | 1 | 19 | - |
|  | *BlaTEM* (-) | 0 | 0 | 0 |  |
| Cephalothin | *BlaTEM* (+) | 0 | 0 | 20 | - |
|  | *BlaTEM* (-) | 0 | 0 | 0 |  |
| Imipenem | *BlaTEM* (+) | 17 | 2 | 1 | - |
|  | *BlaTEM* (-) | 0 | 0 | 0 |  |
| Ciprofloxacin | *qnrA* (+) | 3 | 1 | 5 | 0.218 |
|  | *qnrA* (-) | 7 | 2 | 2 |  |
| Norfloxacin | *qnrA* (+) | 2 | 1 | 6 | 0.205 |
|  | *qnrA* (-) | 6 | 2 | 3 |  |
| Nalidixic acid | *qnrA* (+) | 1 | 1 | 7 | 0.269 |
|  | *qnrA* (-) | 4 | 0 | 7 |  |
| Trimethoprim/ Sulphamethoxazole | *Sul1* (+) | 2 | 2 | 11 | 0.352 |
|  | *Sul1* (-) | 2 | 1 | 2 |  |
| Oxytetracycline | *tetA* (+) | 0 | 0 | 11 | - |
|  | *tetA* (-) | 0 | 0 | 9 |  |

**Supplementary Table 3: Genotypic antimicrobial resistance and biofilm production association (n=20)**

| **Antimicrobial Resistance gene** | **Biofilm production** | | | | **p value** |
| --- | --- | --- | --- | --- | --- |
|  | **Strong biofilm producers**  **(n)** | **Moderate biofilm producers (n)** | **Weak biofilm producers (n)** | **Non-biofilm producers (n)** |  |
| *BlaTEM* (+)  *BlaTEM* (-) | 8  0 | 10  0 | 2  0 | 0  0 | - |
| *qnrA* (+)  *qnrA* (-) | 5  3 | 3  7 | 1  1 | 0  0 | 0.383 |
| *Sul1* (+)  *Sul1* (-) | 6  2 | 9  1 | 0  2 | 0  0 | **0.027** |
| *tetA* (+)  *tetA* (-) | 7  1 | 4  6 | 0  2 | 0  0 | **0.034** |

**Supplementary Table 4: Class 1 integron and genotypic antimicrobial resistance association (n=20)**

| **Antimicrobial Resistance Gene** | **Class 1 Integron Gene** | | **p value** |
| --- | --- | --- | --- |
|  | **(-) (n)** | **(+) (n)** |  |
| *BlaTEM* (+)  *BlaTEM* (-) | 9  0 | 1  0 | - |
| *qnrA* (+)  *qnrA* (-) | 1  8 | 8  3 | **0.006** |
| *Sul1* (+)  *Sul1* (-) | 7  2 | 8  3 | 0.795 |
| *tetA* (+)  *tetA* (-) | 4  5 | 7  4 | 0.391 |

**Supplementary Table 5: Class 2 integron and genotypic antimicrobial resistance association (n=20)**

| **Antimicrobial Resistance Gene** | **Class 2 Integron Gene** | | **p value** |
| --- | --- | --- | --- |
|  | **(-) (n)** | **(+) (n)** |  |
| *BlaTEM* (+)  *BlaTEM* (-) | 20  0 | 0  0 | - |
| *qnrA* (+)  *qnrA* (-) | 9  11 | 0  0 | **-** |
| *Sul1* (+)  *Sul1* (-) | 15  5 | 0  0 | - |
| *tetA* (+)  *tetA* (-) | 11  9 | 0  0 | - |

**Supplementary Figures**


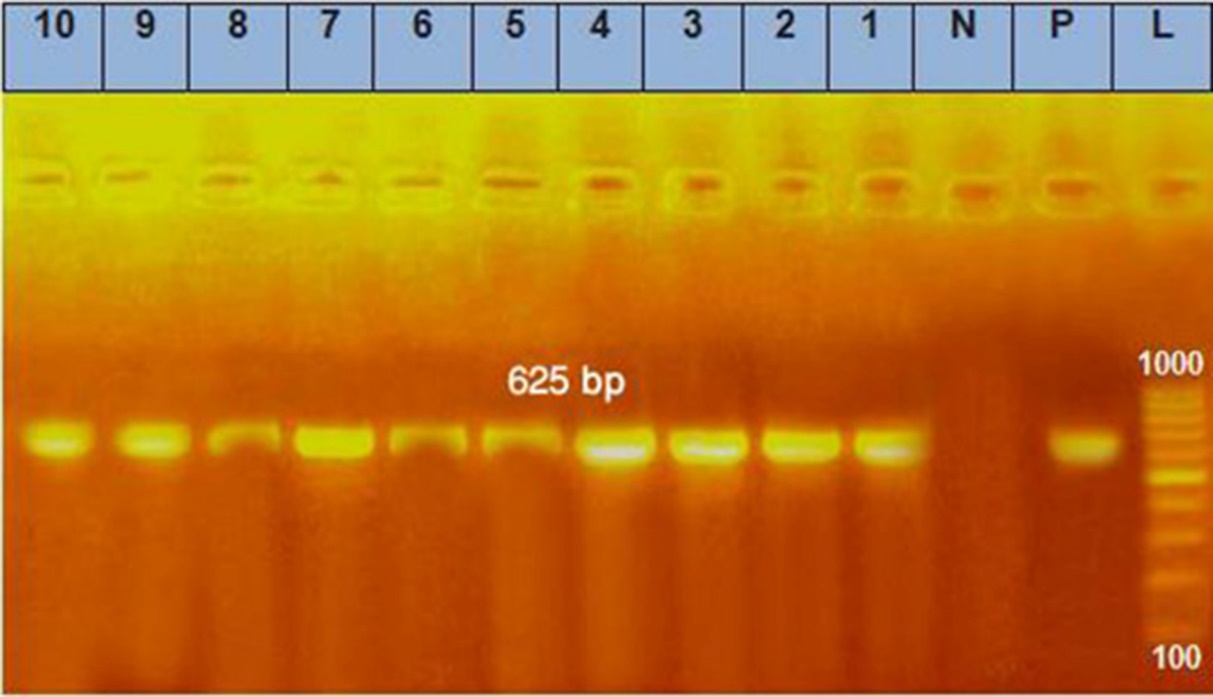


**Supplementary Figure 1.** Agarose gel electrophoresis for PCR products that targeted *16S* *rRNA* gene in *A*. *hydrophila* isolates. Lane L: DNA ladder (100-1000 bp), Lane N: Negative control, Lane P: Positive control, and Lanes 1-10: PCR products of *A*. *hydrophila* isolates (*C. gariepinus*: Isolates 1, 6, 7, and 9; *O. niloticus*: Isolates 2-5, 8, and 10) with specific size showing positive bands at 625-bp in all the examined isolates.


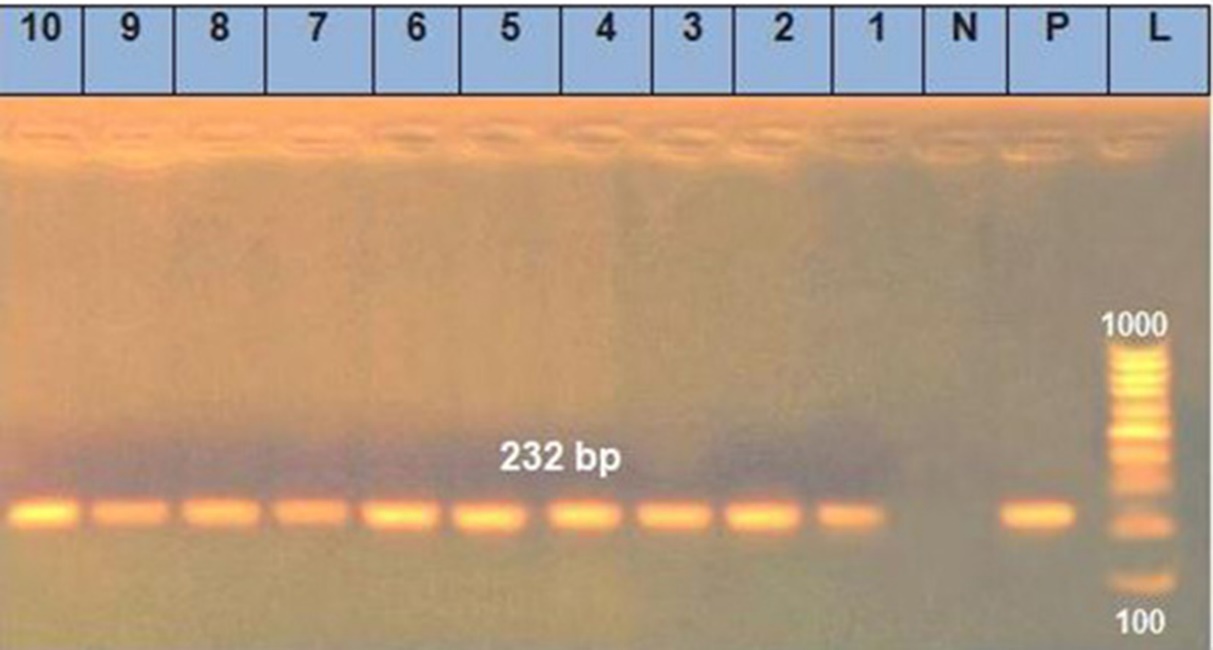


**Supplementary Figure 2.** Agarose gel electrophoresis for PCR products that targeted *act* gene in *A*. *hydrophila* isolates. Lane L: DNA ladder (100-1000 bp), Lane N: Negative control, Lane P: Positive control, and Lanes 1-10: PCR products of *A*. *hydrophila* isolates with specific size showing positive bands at 232-bp in all the examined isolates described in Supplementary Figure 1.


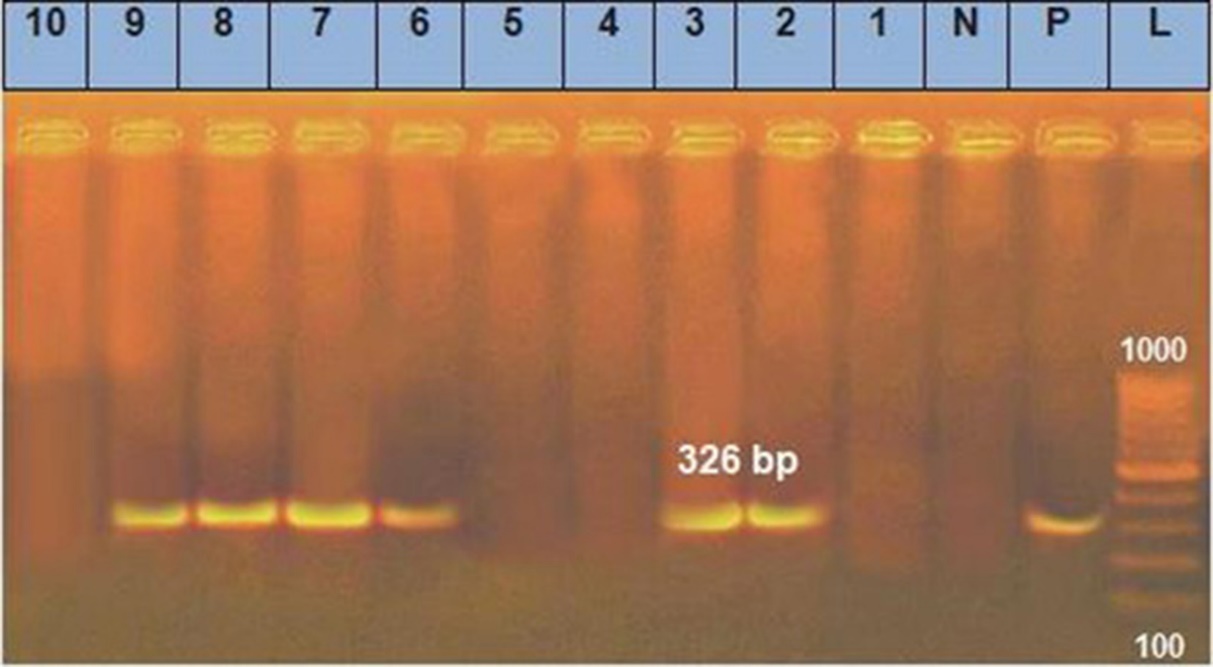


**Supplementary Figure 3.** Agarose gel electrophoresis for PCR products that targeted *aer* gene in *A*. *hydrophila* isolates. Lane L: DNA ladder (100-1000 bp), Lane N: Negative control, Lane P: Positive control, and Lanes 1-10: PCR products of *A*. *hydrophila* isolates with specific size showing positive bands at 326-bp in isolates 2, 3, 6, 7, 8 and 9 described in Supplementary Figure 1.


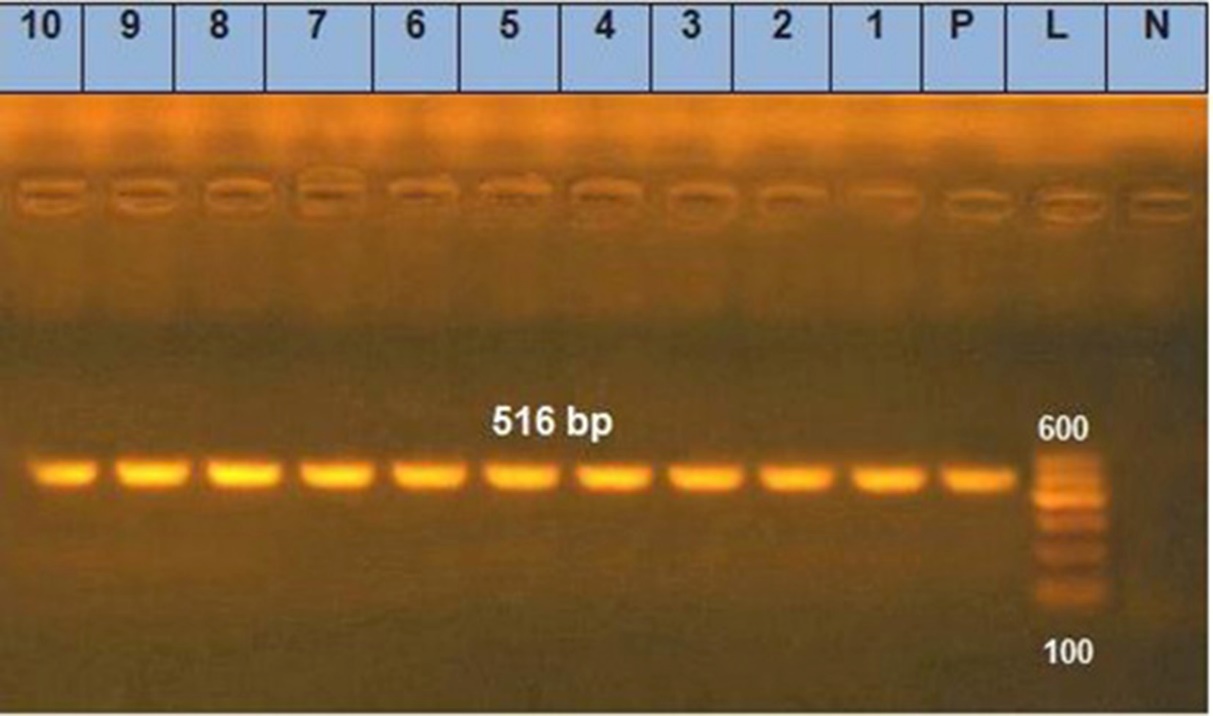


**Supplementary Figure 4.** Agarose gel electrophoresis for PCR products that targeted *blaTEM* gene in *A*. *hydrophila* isolates. Lane L: DNA ladder (100-600 bp), Lane N: Negative control, Lane P: Positive control, and Lanes 1-10: PCR products of *A*. *hydrophila* isolates with specific size showing positive bands at 516-bp in all the examined isolates described in Supplementary Figure 1.


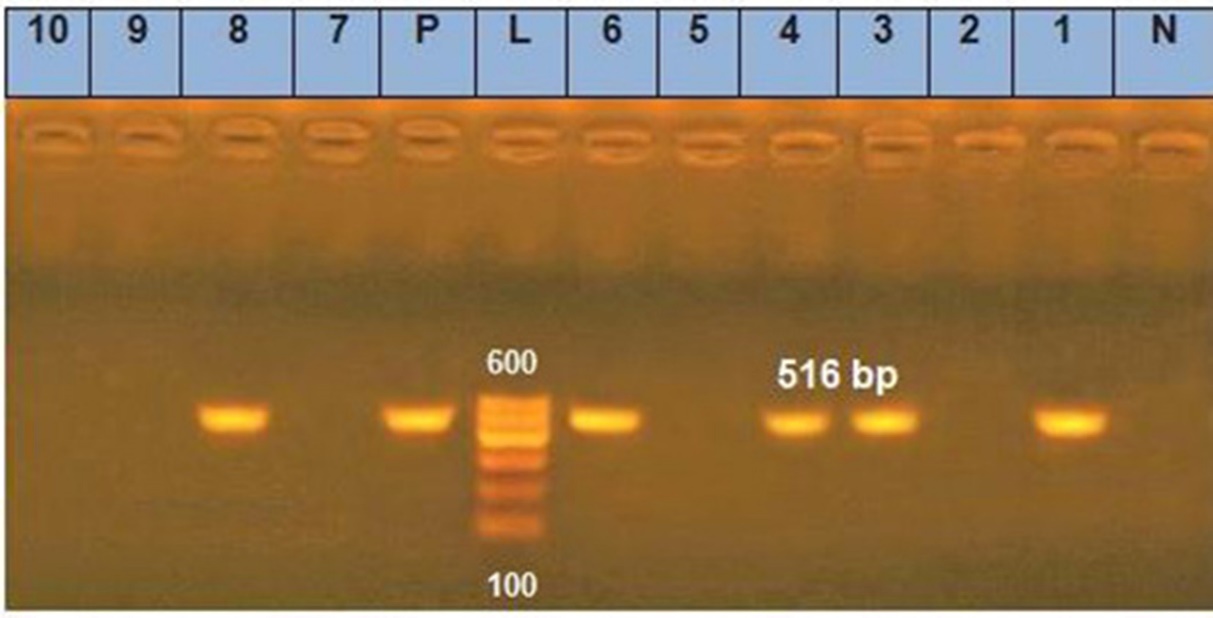


**Supplementary Figure 5.** Agarose gel electrophoresis for PCR products that targeted *qnrA* gene in *A*. *hydrophila* isolates. Lane L: DNA ladder (100-600 bp), Lane N: Negative control, Lane P: Positive control, and Lanes 1-10: PCR products of *A*. *hydrophila* isolates with specific size showing positive bands at 516-bp in isolates 1, 3, 4, 6 and 8 described in Supplementary Figure 1.


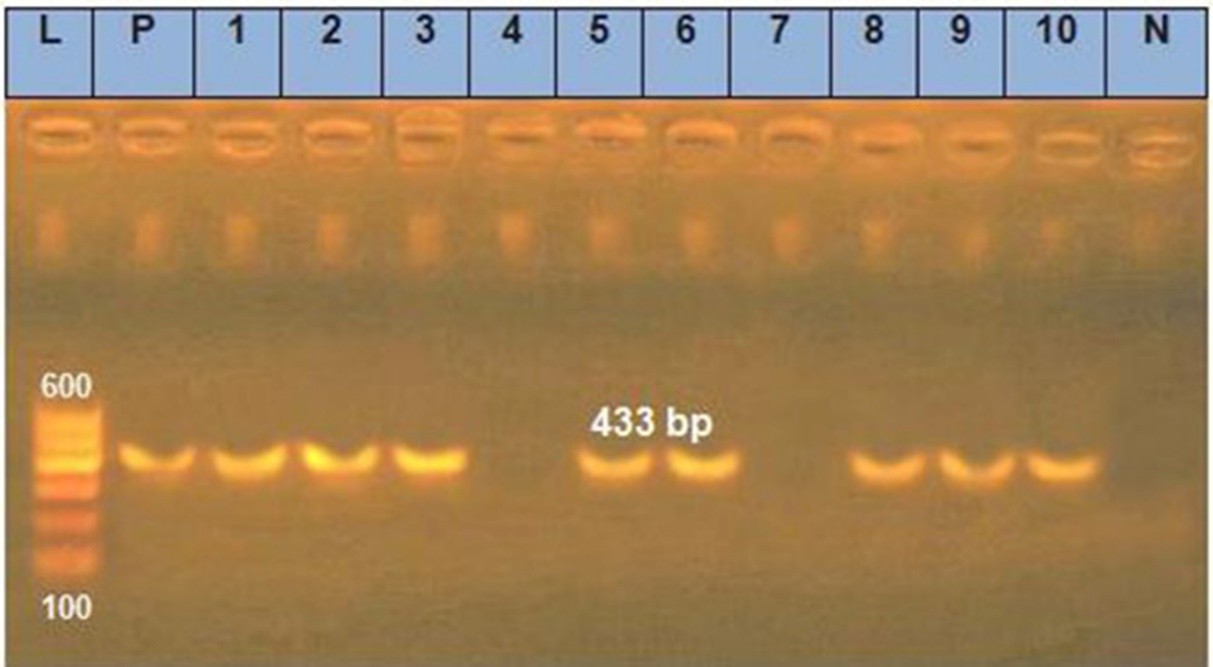


**Supplementary Figure 6.** Agarose gel electrophoresis for PCR products that targeted *sul1* gene in *A*. *hydrophila* isolates. Lane L: DNA ladder (100-600 bp), Lane N: Negative control, Lane P: Positive control, and Lanes 1-10: PCR products of *A*. *hydrophila* isolates with specific size showing positive bands at 433-bp in isolates 1, 2, 3, 5, 6, 8, 9 and 10 described in Supplementary Figure 1.


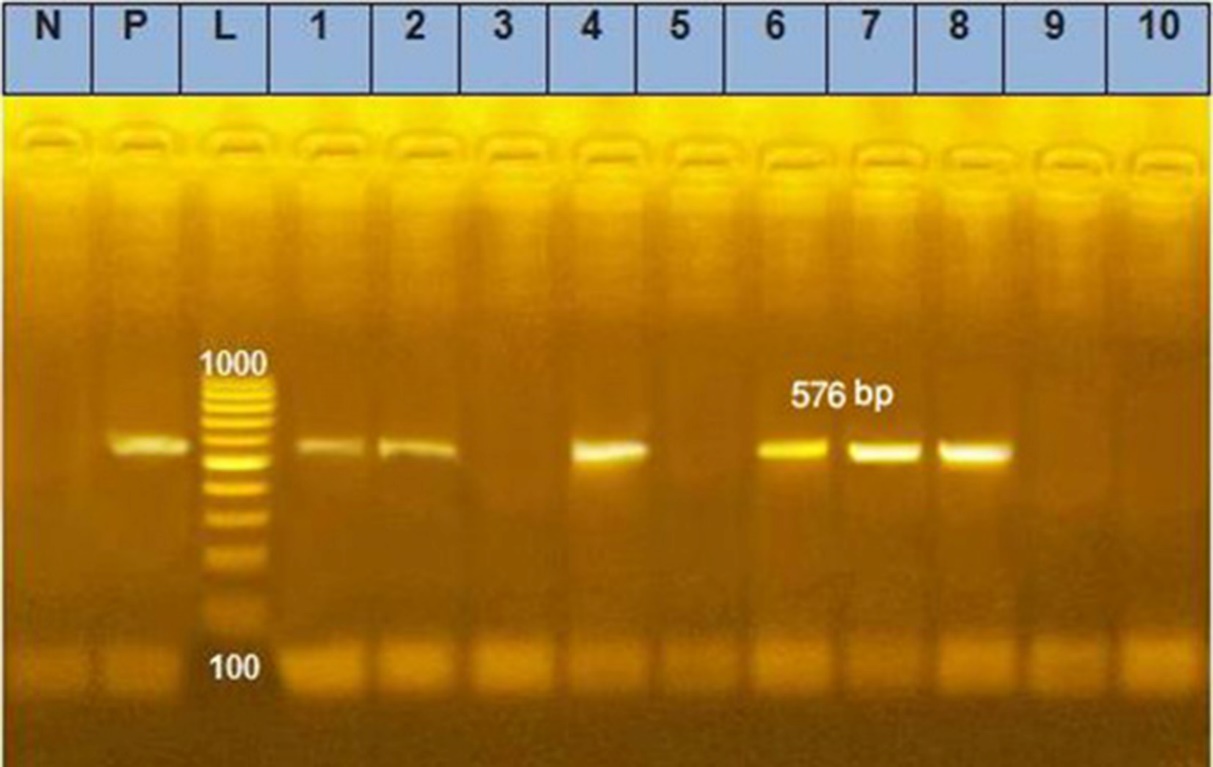


**Supplementary Figure 7.** Agarose gel electrophoresis for PCR products that targeted *tetA* gene in *A*. *hydrophila* isolates. Lane L: DNA ladder (100-1000 bp), Lane N: Negative control, Lane P: Positive control, and Lanes 1-10: PCR products of *A*. *hydrophila* isolates with specific size showing positive bands at 576-bp in isolates 1, 2, 4, 6, 7 and 8 described in Supplementary Figure 1.


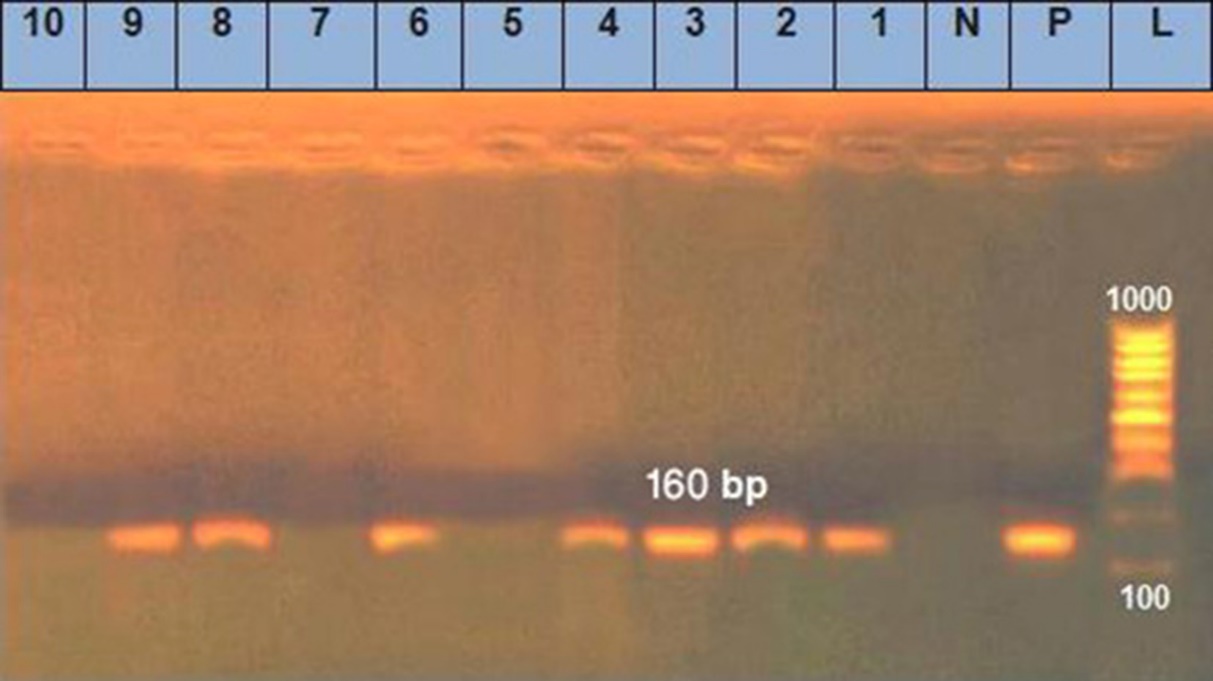


**Supplementary Figure 8.** Agarose gel electrophoresis for PCR products that targeted *int1* gene in *A*. *hydrophila* isolates. Lane L: DNA ladder (100-1000 bp), Lane N: Negative control, Lane P: Positive control, and Lanes 1-10: PCR products of *A*. *hydrophila* isolates with specific size showing positive bands at 160-bp in isolates 1, 2, 3, 4, 6, 8 and 9 described in Supplementary Figure 1.


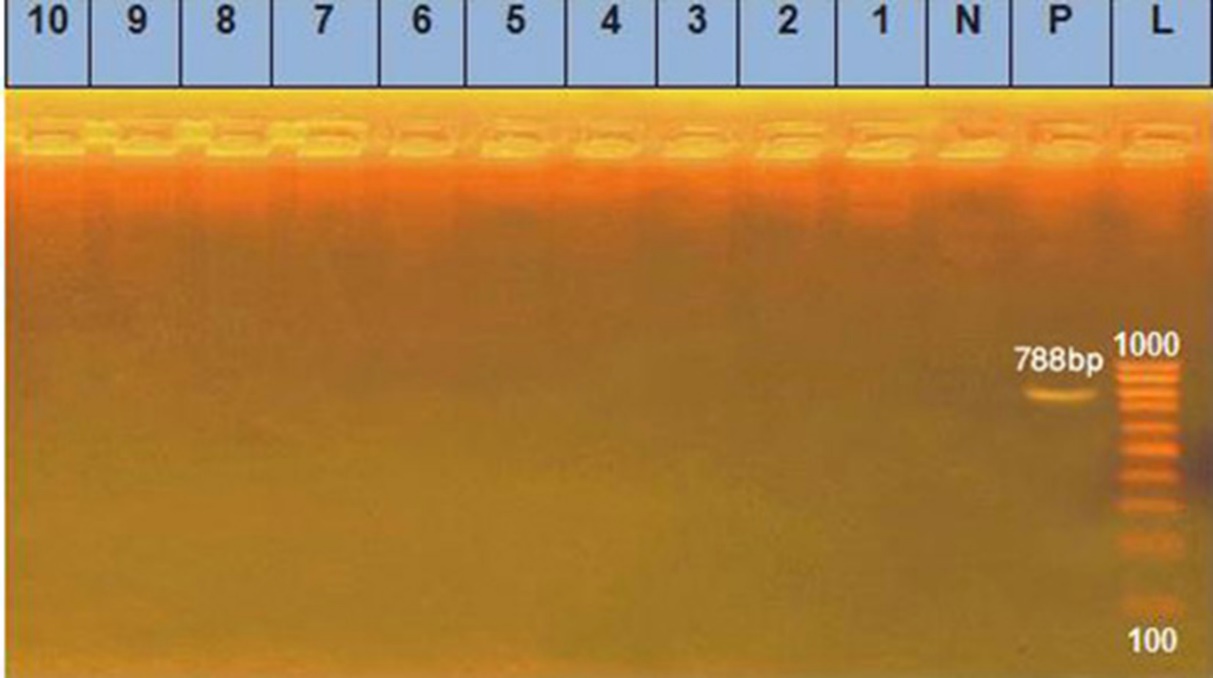


**Supplementary Figure 9.** Agarose gel electrophoresis for PCR products that targeted *int2* gene in *A*. *hydrophila* isolates. Lane L: DNA ladder (100-1000 bp), Lane N: Negative control, Lane P: Positive control, and Lanes 1-10: PCR products of *A*. *hydrophila* isolates with specific size showing negative results in all the examined isolates described in Supplementary Figure 1.
